# DNA walking system integrated with enzymatic cleavage reaction for sensitive surface plasmon resonance detection of miRNA

**DOI:** 10.1038/s41598-022-20453-8

**Published:** 2022-09-27

**Authors:** Sijia Chen, Yuhan He, Lin Liu, Jianxiu Wang, Xinyao Yi

**Affiliations:** 1 Hunan Provincial Key Laboratory of Micro & Nano Materials Interface Science, College of Chemistry and Chemical Engineering, Central South University, Changsha, 410083 Hunan People’s Republic of China; 2 Henan Province of Key Laboratory of New Optoelectronic Functional Materials, Anyang Normal University, Anyang, 455000 Henan People’s Republic of China

**Keywords:** Analytical chemistry, Bioanalytical chemistry, Sensors

## Abstract

Abnormal expression levels of miRNA are associated with various tumor diseases, for example, glioma tumors are characterized by the up-regulation of miRNA-182. Surface plasmon resonance (SPR) assay for miRNA-182 from glioma patients was performed via DNA walking amplification strategy. The duplex between aminated swing arm DNA (swDNA) and block DNA (blDNA), and aminated track DNA (trDNA) with a biotin tag were tethered on the poly(ethylene glycol) (PEG)-modified chips. Upon formation of miRNA/blDNA duplex, the SPR signal decreased with the walking process of swDNA, as the biotinylated fragment of trDNA (biotin-TTGGAGT) was detached from the sensor surface caused by the nicking endonuclease Nb.BbvCI. Such a repeated hybridization and cleavage cycle occurred continuously and the detachment of more biotinylated fragments of trDNA from the chips led to the attachment of fewer streptavidin (SA) molecules and then smaller SPR signals. MiRNA-182 with concentrations ranging from 5.0 fM to 1.0 pM could be readily determined and a detection limit of 0.62 fM was achieved. The proposed method was highly selective and possessed remarkable capability for evaluating the expression levels of miRNA-182 in serum samples from healthy donors and glioma patients. The sensing protocol holds great promise for early diagnosis of cancer patients.

## Introduction

As a kind of non-coding and single-stranded RNAs with 18–25 nucleotides, microRNAs (miRNAs) participated in the regulation of gene expression in a large number of biological processes. Abnormal miRNA expression was closely related to cancers^1–3^, cardiovascular diseases^4^, and neurological diseases^5^. However, the detection of miRNAs was challenged by low expression level, short sequence length, ease of degradation, and high sequence similarity.

Traditional methods for miRNA assay included but not limited to microarray^6^, northern blotting^7^, and quantitative real-time polymerase chain reaction (qRT-PCR)^8^. However, these methods either suffered from low sensitivity, or possessed the drawbacks, such as time consumption, and operation complexity. To overcome these problems, the strategies for miRNA assay based on surface plasmon resonance (SPR)^9–23^, electrochemistry^24–27^, fluorescence^28–30^, electrochemical luminescence^31–33^, and colorimetry^34–36^ have emerged. As an optical technique, SPR is capable of measuring the changes in refractive index or thickness occurring at the surface of a metal film (typically gold)^37^, being advantageous over other techniques due to its label-free, real-time, and highly sensitive properties^37^. Typically, the SPR signal can be amplified through recognition by antibodies^9^ and nanoparticles^10,12,13^, or biotin-streptavidin complexation^10,14^, catalytic hairpin assembly^15–17^, and enzymatic reaction^18^. Metal nanoparticles have been widely applied for SPR detection of miRNA. For example, based on DNA amplification and adsorption of silver nanoparticles (AgNPs), enzyme-free SPR assay of miRNA with a detection limit of 0.6 fM has been carried out^11^. In situ generated AgNPs via hybridization chain reaction were involved for amplified SPR detection of let-7a miRNA^12^. Wang et al. have proposed a SPR platform for miRNA-21 assay by coupling gold nanoparticles (AuNPs) with super sandwich assembly^19^. Furthermore, the same group constructed graphene oxide-AuNPs hybrids for SPR detection of miRNA-141 extracted from human cancer cell lines^20^. Although the metal nanoparticles with high molecular weight and the electronic coupling between metal nanoparticles and the Au film could effectively enhance the SPR signals^38,39^, the nonspecific adsorption of metal nanoparticles limited their further applications in SPR. SA is widely used to amplify the SPR signals due to its large molecular weight (56 kDa) and strong binding with biotin (K_D_ = 10^–15^ M^−1^) ^40^.

DNA nanotechnology-based systems, such as HCR^41^, rolling circle amplification (RCA)^42^, and DNA walking systems^43^ have emerged for biosensing due to easy fabrication, low cost, and good stability^44^. The DNA walking system consisting of a swing arm, a track, and a trigger has attracted much attention^45^. Typically, the target triggered the DNA walking mechanism and the swing arm moved automatically along the track, leading to cascade signal amplification. Toehold-mediated strand displacement reaction (TSDR) served as a promising alternative for detection of single-nucleotide polymorphisms due to its high selectivity^46,47^. To perform highly sensitive and specific SPR assay, combining TSDR, DNA walking process and enzymatic cleavage reaction might be a better choice.

Herein, sensitive and selective detection of miRNA was achieved using SPR. In the presence of miRNA-182, the SPR signal decreased with the walking process, which was caused by the detachment of the biotinylated fragments of trDNA (biotin-TTGGAGT) from the sensor surface with the help of Nb.BbvCI. The proposed method holds great promise for early diagnosis of miRNA-related major diseases.

## Experimental

### Materials

Triethylene glycol mono-11-mercaptoundecyl ether (HSC_11_PEG_3_-OH), ethanolamine hydrochloride (EA), *N*-(3-dimethylaminopropyl)-*N*′-ethylcarbodiimide hydrochloride (EDC), *N*-hydroxysuccinimide (NHS) and streptavidin (SA) were purchased from Sigma-Aldrich (St. Louis, MO). Hexaethylene glycol mono-11-mercaptoundecyl acid (HSC_11_PEG_6_-COOH) was acquired from Dojindo Laboratories (Kumamoto, Japan). Anhydrous ethanol, NaCl, MgCl_2_, K_2_HPO_4_, KH_2_PO_4_, agarose, DNA marker, 6× loading buffer, and diethylpyrocarbonate (DEPC)-treated deionized water were obtained from Sangon Biotech Co., Ltd. (Shanghai, China). The nicking endonuclease Nb.BbvCI was obtained from New England Biolabs (Ipswich, MA). High performance liquid chromatography (HPLC)-purified miRNAs and DNAs were synthesized by Sangon Biotech Co., Ltd. (Shanghai, China) and their sequences were listed in Table 1 (mismatched bases relative to that of miRNA-182 were underlined). Other reagents were of analytical purity and used as received. All solutions were prepared with deionized water treated with a water purification system (Simplicity185, Millipore Corp., Billerica, MA).

**Table 1:** Sequences of DNAs and miRNAs.

Oligonucleotides	Sequences (from 5′ to 3′)
Swing arm strand (sw DNA)	NH_2_-T_40_-GGTAGAACTCACACTCCTCAGC
Track (trDNA)	NH_2_-T_10_-GCTGAGGTT-biotin
Block (blDNA)	TGAGGAGTGTGAGTTCTACCATTGCCAAA
miRNA-182	UUUGGCAAUGGUAGAACUCACACU
miRNA-96	UUUGGCACUAGCACAUUUUUGCU
miRNA-182-3p	UGGUUCUAGACUUGCCAACUA
miRNA-155	UUAAUGCUAAUCGUGAUAGGGGUU

### Procedures

#### Solution preparation

HSC_11_PEG_6_-COOH and HSC_11_PEG_3_-OH were dissolved in anhydrous ethanol. The mixed solution of 0.4 M EDC and 0.1 M NHS in deionized water was used to activate the carboxylic acid groups on PEG chips. The swDNA/blDNA duplex was formed by mixing swDNA and blDNA in phosphate-buffered saline (PBS, pH 7.4, 10 mM phosphate/10 mM NaCl) at a molar ratio of 1:2, heating to 95 °C for 5 min and then cooling down to room temperature. To maintain an RNase-free environment, the miRNAs were dissolved in PBS containing 5 mM MgCl_2_ that was prepared with DEPC-treated deionized water. The nicking endonuclease Nb.BbvCI was diluted with 1× CutSmart buffer (pH 7.9) containing 50 mM potassium acetate, 20 mM tris–acetate, 10 mM magnesium acetate and 100 μg/mL bovine serum albumin. SA was diluted to 50 nM with PBS.

#### Chip modification and SPR detection

The PEG-covered chips were formed by immersing the cleaned Au films in a mixed solution of HSC_11_PEG_6_-COOH (0.2 mM) and HSC_11_PEG_3_-OH (1.8 mM) in the dark for 48 h. Via activation of the carboxylic acid groups at the PEG chain end with EDC/NHS for 30 min at room temperature, the aminated swDNA/blDNA duplex and aminated trDNA with a molar ratio of 1:100 were anchored on the PEG chips through amide bond formation. After washing thoroughly with PBS and deionized water, the sensor chips were soaked in 0.1 M EA for 10 min to block the unreacted sites. Serum samples or miRNA with various concentrations were cast onto the chips for 3 h at 37 °C. After completion of the cleavage reaction by 50 U/mL of Nb.BbvCI for 2 h at 37 °C, the sensor chips were mounted onto the BI-4000 SPR instrument (Biosensing Instrument Inc., Tempe, AZ). Finally, 50 nM SA solution was preloaded into a 200 μL-sample loop and delivered to the sensor chips by the carrier solution at a flow rate of 20 μL/min. The degassed PBS that was treated by vacuum pumping for 30 min served as the carrier solution.

#### Agarose gel electrophoresis

Agarose gels (4%) were prepared as reported^48^. Briefly, 2.0 g agarose was dissolved in 50 mL 1× Tris‐acetate‐EDTA (TAE) buffer with the aid of microwave oven. Afterwards, 2.5 μL nucleic acid stain was evenly mixed with the slightly cooled agarose solution, followed by pouring into the gel mold. The analytes were loaded onto the gels in 6× DNA loading buffer. Gel electrophoresis was run in 1× TAE buffer at 120 V for 1 h on a Mini-Sub Cell GT electrophoresis system (Bio-Rad Laboratories, Hercules, CA), and imaged on a GelView 1500Plus intelligent gel imaging system (Guangzhou, China).

## Results and discussion

**Figure 1:**
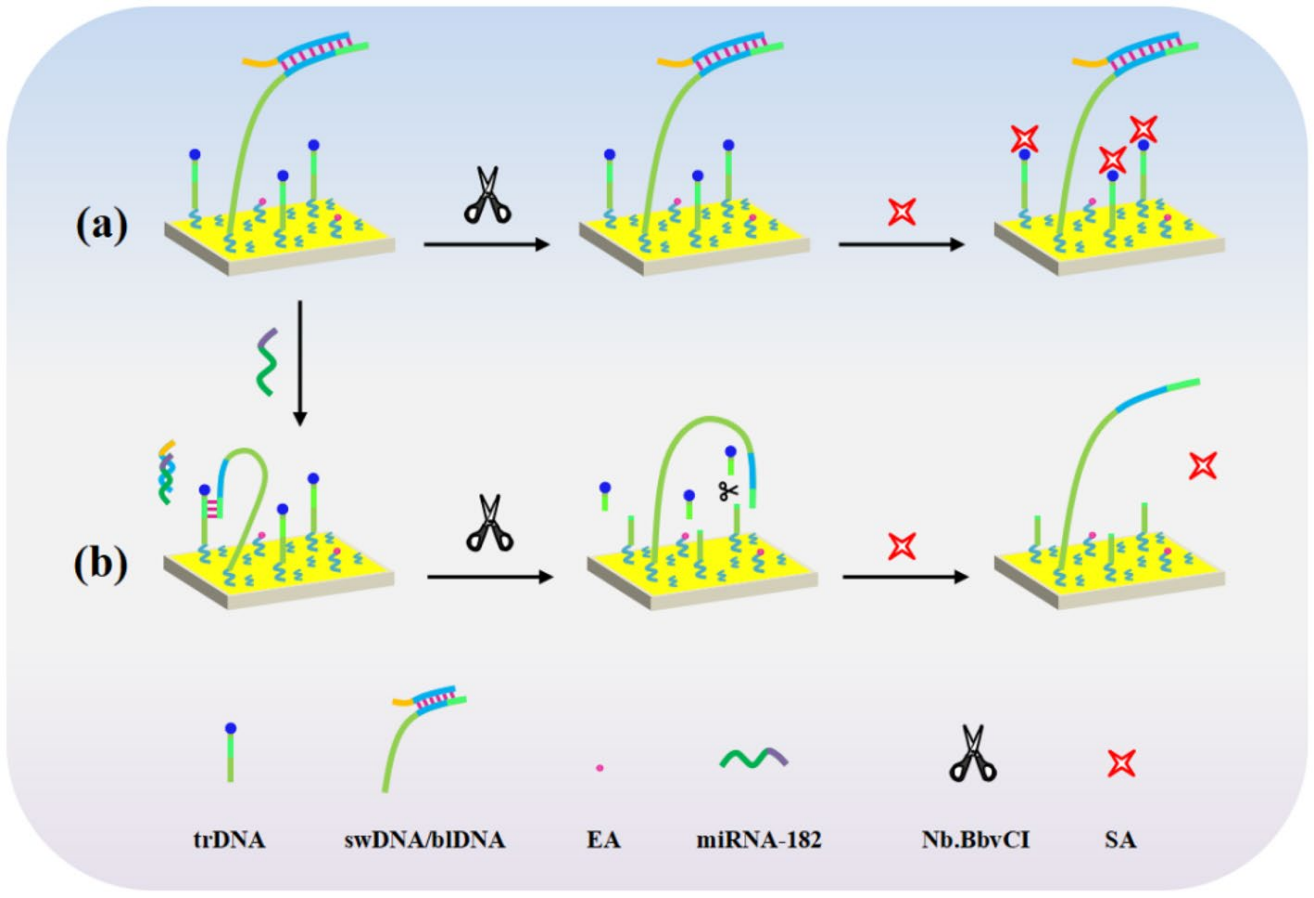
Schematic representation of SPR assay of miRNA based on DNA walking process integrated with enzymatic cleavage reaction.

The principle for SPR assay of miRNA based on DNA walking process and enzymatic cleavage reaction was depicted in Fig. 1. The aminated swDNA/blDNA duplex and aminated trDNA with a biotin tag were tethered on the PEG-modified chips through EDC/NHS coupling chemistry. The blDNA contained an exposed 9-nt toehold domain (orange color), which was complementary to the sequence of target miRNA. In the absence of miRNA-182, the biotin tag on the surface-confined trDNA remained intact and could be recognized by SA (56 KDa)^40^. Consequently, the large mass of SA amplified the SPR signals (a). However, the existence of miRNA triggered the TSDR, leading to the release of swDNA by forming blDNA/miRNA duplex. The released swDNA was then hybridized with the surface-confined trDNA and the biotinylated fragment on trDNA (biotin-TTGGAGT) could be cleaved by nicking endonuclease Nb.BbvCI with high sequence specificity (3′-GGAGT↓CG-5′)^49–51^. The released swDNA then autonomously walked on the chip surface, and with the help of Nb.BbvCI, more biotinylated fragments of trDNA were detached from the chips. As a result, the attachment of SA was remarkably hindered, leading to smaller SPR signals (b).

**Figure 2:**
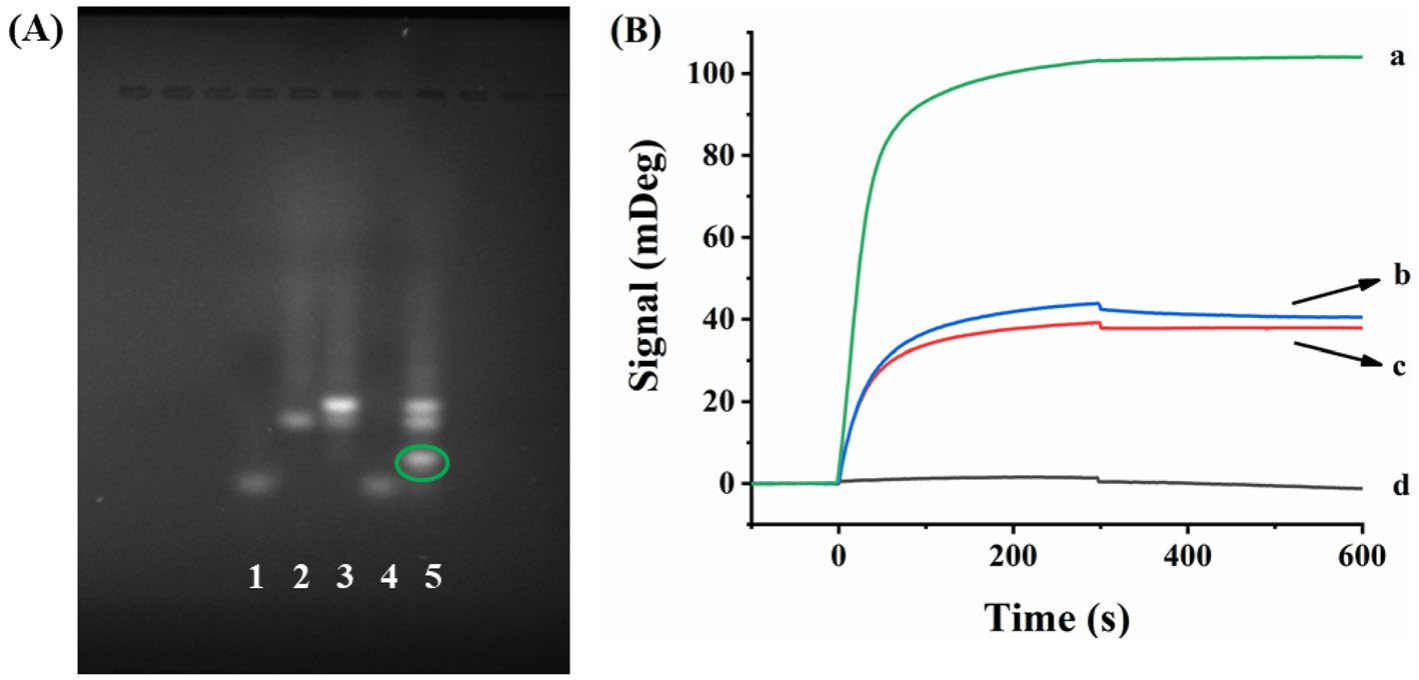
(**A**) Gel electrophoresis characterization of the TSDR. Lane 1: 2 μM blDNA; Lane 2: 1 μM swDNA; Lane 3: 1 μM swDNA/2 μM blDNA duplex; Lane 4: 2 μM miRNA-182; Lane 5: 1 μM swDNA/2 μM blDNA duplex + 2 μM miRNA-182. (**B**) SPR sensorgrams upon injection of 50 nM SA onto the Nb.BbvCI-treated fluidic channels that were covered with (a) trDNA + swDNA/blDNA duplex; (b) trDNA + swDNA/blDNA + miRNA-182; (c) trDNA + swDNA; (d) non-biotinylated trDNA + swDNA/blDNA duplex + miRNA-182.

The occurrence of TSDR was confirmed by gel electrophoresis (Fig. 2A). The single band with different migration rates in lane 1, lane 2 and lane 4 corresponded to blDNA, swDNA and miRNA. After hybridization with blDNA, a new band with slower migration appeared, indicating the swDNA/blDNA duplex formation (top band in lane 3). The existence of miRNA triggered the walking mechanism, and the formation of blDNA/miRNA duplex was evidenced by a new band in lane 5 (surrounded by green circle). In the same time, the color of the band corresponding to swDNA deepened (middle band in lane 5), which demonstrates that the swDNA was successfully released. Gel electrophoresis assay of the DNA chains after enzymatic cleavage reaction was performed (Fig. S1). The single bands in lane 1 and lane 2 corresponded to trDNA and swDNA, respectively. Via hybridization between swDNA and trDNA, the bright band in lane 3 indicates the formation of the swDNA/trDNA duplex (lane 3). After the enzymatic cleavage reaction, the trDNA was broken into two fragments of TTTTTTTTTTGC (blue square, lane 4) and TGAGGTT (too small to be seen).

Via combining DNA walking process and enzymatic cleavage reaction, SPR assay of miRNA was conducted (Fig. 2B). In the absence of miRNA, the protection of swDNA by blDNA hindered the occurrence of TSDR, and the recognition of the biotinylated trDNA by SA produced a large SPR signal of 104 mDeg (curve a, Fig. 2B). However, the existence of miRNA causes miRNA/blDNA duplex formation and the subsequent release of swDNA, triggered the walking mechanism. The released swDNA then hybridized with the surface-confined trDNA and the biotinylated fragment of trDNA could be cleaved by Nb.BbvCI. With the release of more swDNA, large numbers of trDNAs were enzymatically cleaved. As a result, the incorporation of SA led to an SPR signal of 41 mDeg (curve b, Fig. 2B). Note that the aminated swDNA/blDNA duplex and aminated trDNA were simultaneously anchored on the PEG-modified chips, and the molar ratio between swDNA/blDNA duplex and trDNA was optimized. The swDNA/blDNA duplex was first formed through hybridization of 25 nM swDNA with 50 nM blDNA. The resultant duplex was mixed with trDNA with different molar ratios, and then immobilized on the PEG chips. The SPR signals upon injection of 50 nM SA increased with the molar ratio (swDNA:trDNA) in the range of 1:20, 1:40, 1:80, 1:100 and 1:120, and began to level off beyond 1:100 (Fig. S2A). Furthermore, the extent of cleavage reached the maximum at 2 h (Fig. S2B). Thus, swDNA (25 nM)/blDNA (50 nM) duplex and 2.5 μM trDNA were tethered on the PEG chips, and the optimal enzymatic cleavage time was maintained at 2 h. The relatively large SPR signal of 41 mDeg caused by incomplete cleavage reaction (curve b) might be ascribed to the limited walking area of the released swDNAs on the two-dimensional sensor surface, as evidenced by the similar SPR signal in the case of unprotected swDNA and trDNA (curve c, Fig. 2B). When the biotinylated trDNAs in curve b were replaced by the non-biotinylated ones, almost no SPR signal was obtained upon injection of 50 nM SA (curve d, Fig. 2B), indicating that the nonspecific adsorption of SA on the chip surface was negligible.

**Figure 3:**
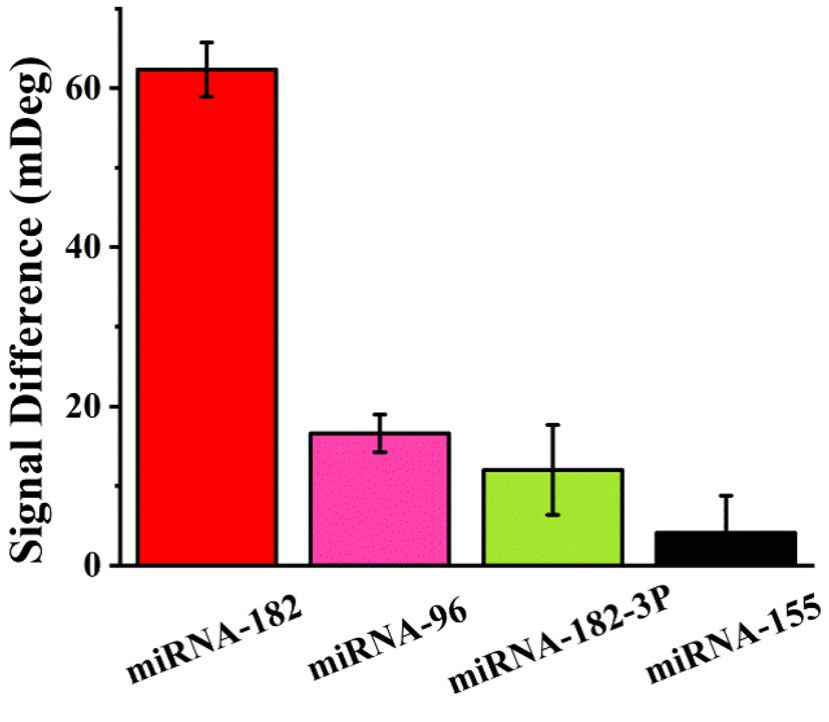
Selectivity of the SPR method for miRNA-182 assay. The concentration of the various miRNA species was maintained at 50 pM. The error bars indicated the standard deviation of three repeated measurements.

The selectivity of the method for miRNA-182 assay was assessed by examining several miRNA-182 family members (miRNA-96, miRNA-182-3p)^52,53^ and other miRNA sequence (miRNA-155) (Fig. 3). The signal difference in the vertical axis in Fig. 3 was defined by subtracting the SPR signals in the case of different miRNAs from the blank (curve a in Fig. 2B). MiRNA-96, miRNA-182-3p and miRNA-155 possessed 11, 15 and 20 mismatched bases related to miRNA-182, respectively. In the presence of target miRNA-182, the SPR signal difference was 62 mDeg, suggesting the occurrence of the enzymatic cleavage reaction. However, in the cases of miRNA-96, miRNA-182-3p and miRNA-155, the hybridization between miRNA and blRNA was hindered to a larger extent, and the SPR signal differences of 17 mDeg, 12 mDeg and 4 mDeg were attained, respectively. The results demonstrated the capability of the method for distinguishing miRNA-182 from other similar sequences.

**Figure 4:**
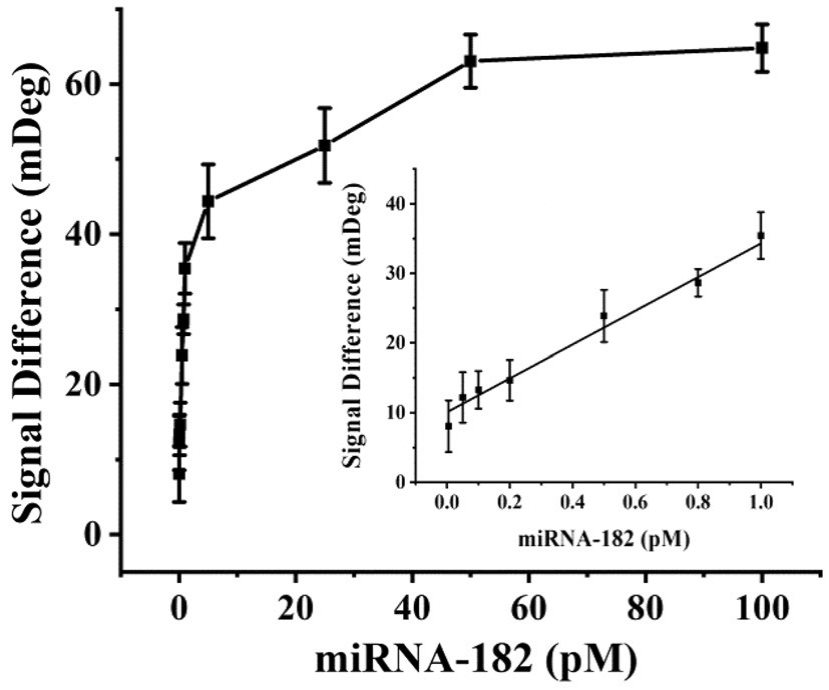
Dependence of the SPR signal difference on the concentrations of miRNA-182. The inset showed the linear portion of the curve with concentrations ranging from 0.005 to 1.0 pM. The error bars represented the standard deviations for three replicate measurements.

The dependence of SPR signals on the concentrations of miRNA-182 was investigated (Fig. 4, Fig. S3). The signal difference, defined as that in Fig. 3, was linearly proportional to miRNA concentrations ranging from 0.005 pM to 1.0 pM. The linear regression equation was expressed as Signal Difference (mDeg) = 24.20 [miRNA-182] (pM) + 10.08 (*R*^2^ = 0.985). The limit of detection (LOD) was estimated to be 0.62 fM based on 3 SD/Slope, where SD was the standard deviation of the blank measurements in the absence of miRNA-182. The relative standard deviations (RSDs) for three replicate measurements of all the miRNA concentrations were below 9%, indicating high reproducibility of the assay. The analytical performances of the proposed method were comparable with those of other signal-amplified SPR methods and DNA walking-based amplification methods (Table 2).

**Table 2:** Comparison of the analytical performances of the proposed method for miRNA assay with those of other signal-amplified SPR methods^10,12,13,17^ and DNA walking-based amplification methods^25,54–57^.

Analytical method	Amplification strategy	LOD (fM)	Linear range (pM)	Reference
SPR	Streptavidinylated Au nanorods	45	0.1–100	^10^
SPR	Silver nanoparticles	0.35	0.001–0.1	^12^
SPR	Gold nanoparticles	0.045	20–10,000	^13^
SPR	Catalytic hairpin assembly	53.7	0.1–1500	^17^
Electrochemistry	Enzyme-free target recycling	0.31	0.001–10,000	^25^
Electrochemistry	HCR-DNAzyme cascade amplification	0.02	0.0001–100,000	^54^
Electrochemiluminescence	Enzyme-powered cascade amplification	1.5	0.01–100,000	^55^
Fluorescence	Catalytic hairpin assembly	0.043	156–7000	^56^
Colorimetry	Magnetic 3D DNA walker	16.7	0.05–1 and 1–10	^57^
SPR	DNA walking combined with enzymatic cleavage	0.62	0.005–1.0	This work

**Figure 5:**
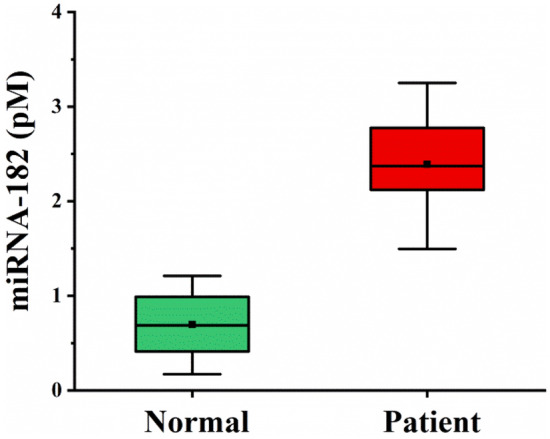
Concentrations of miRNA-182 from sera of three healthy donors and three glioma patients. Each sample was measured three times. The squares indicated the average levels of miRNA-182 from the samples.

The feasibility of the proposed method for determination of the expression levels of miRNA-182 in serum samples from glioma patients and healthy donors has been demonstrated. The serum samples were diluted 5 times with PBS, and then treated by ultrafiltration membranes with a molecular weight cut-off of 10,000 Da to remove high-molecular-weight species. As shown in Fig. 5, the miRNA-182 was highly expressed in glioma patients, and the average expression levels of miRNA-182 from three glioma patients were 3.4 times higher than those from three healthy donors, being consistent with the results by electrochemistry and RT-PCR^58,59^. The SPR method based on DNA walking and enzyme cleavage for miRNA detection served as a promising alternative for point-of-care diagnosis of disease-related biomarkers.

## Conclusion

In summary, SPR assay of miRNA based on DNA walking process and enzymatic cleavage reaction has been developed. The rationally designed protocol possessed high sensitivity and specificity due to the repeated hybridization and cleavage cycles occurring on the chip surface. The capability of the method for differentiating miRNA-182 with different concentrations and miRNAs with different sequences was demonstrated. The method exhibited a detection limit of 0.62 fM and was employed for quantificaiton of miRNA-182 in serum samples. The expression levels of miRNA-182 from glioma patients were 3.4 times higher than those from healthy donors, proving the reliability of the method for complicated samples analysis. The sensing strategy holds great potential for early diagnosis of major clinical diseases.

## Supplementary Information

Supplementary Figures.

## Data Availability

The datasets used and/or analyzed during the current study are available from the corresponding author on reasonable request.
