# Supplementary material for: DNA walking system integrated with enzymatic cleavage reaction for sensitive surface plasmon resonance detection of miRNA
Source: Sci Rep. 2022 Sep 27;12:16093. doi: 10.1038/s41598-022-20453-8 (PMC9515148; doi:10.1038/s41598-022-20453-8)
Supplement: Supplementary file 1 — Supplementary Figures. [file 41598_2022_20453_MOESM1_ESM.docx]

**Supporting Information**

**DNA walking system integrated with enzymatic cleavage reaction for sensitive surface plasmon resonance detection of miRNA**

Sijia Chen^a^, Yuhan He^a^, Lin Liu^b^, Jianxiu Wang*^a^, Xinyao Yi*^a^

^a^Hunan Provincial Key Laboratory of Micro & Nano Materials Interface Science, College of Chemistry and Chemical Engineering, Central South University, Changsha, Hunan 410083, P. R. China

^b^Henan Province of Key Laboratory of New Optoelectronic Functional Materials, Anyang Normal University, Anyang, Henan 455000, P. R. China

*Corresponding Author

Email address: [yixinyao@csu.edu.cn](mailto:yixinyao@csu.edu.cn) (Xinyao Yi); [jxiuwang@csu.edu.cn](mailto:jxiuwang@csu.edu.cn) (Jianxiu Wang)


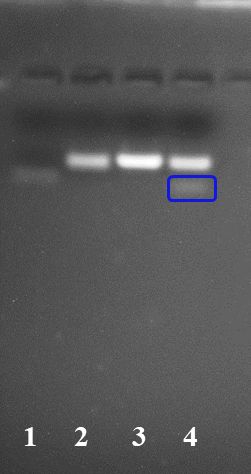


**Figure S1.** Gel electrophoresis characterization of the enzymatic cleavage reaction. Lane 1: 3 μM trDNA; Lane 2: 3 μM swDNA; Lane 3: 3 μM swDNA/3 μM trDNA duplex; Lane 4: 3 μM swDNA/3 μM trDNA duplex + 50 U/mL Nb.BbvCI.


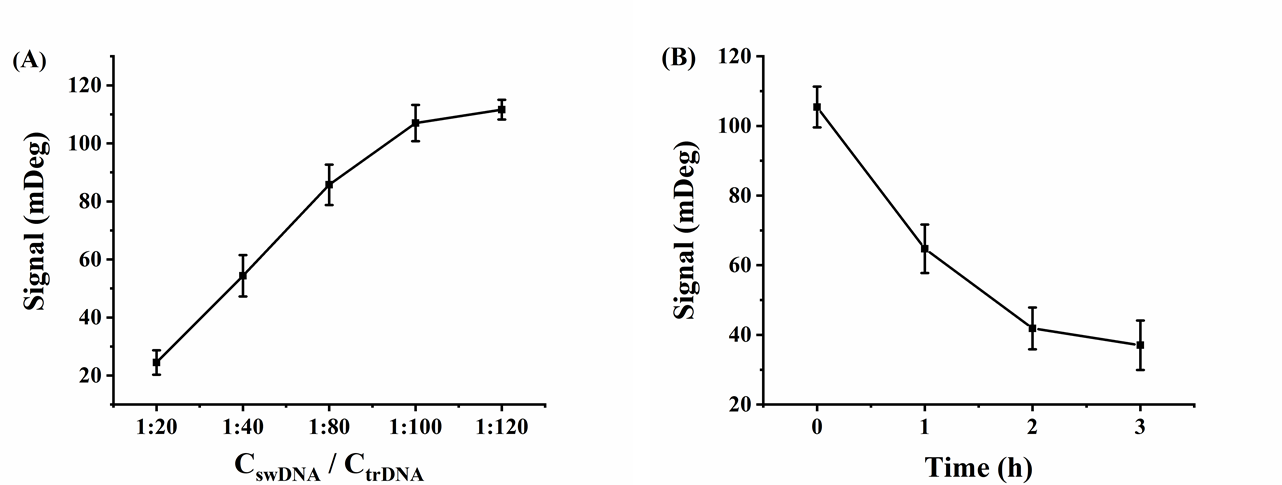


**Figure S2.** Dependence of the SPR signals on the swDNA/trDNA ratio (A) and enzymatic cleavage time (B).





**Figure S3.** SPR sensorgrams upon injection of SA under different miRNA-182 concentrations (from a to j : 0.005 pM, 0.05 pM, 0.1 pM, 0.2 pM, 0.5 pM, 0.8 pM, 1 pM, 5 pM, 25 pM, 50 pM, respectively).
